# Impact of Fibromyalgia on Functioning in Obese Patients Undergoing Comprehensive Rehabilitation

**DOI:** 10.1371/journal.pone.0091392

**Published:** 2014-03-11

**Authors:** Marco Arreghini, Gian Mauro Manzoni, Gianluca Castelnuovo, Cristina Santovito, Paolo Capodaglio

**Affiliations:** 1 Rehabilitation Unit, Istituto Auxologico Italiano IRCCS, Piancavallo (VB), Italy; 2 Psychology Research Laboratory, Istituto Auxologico Italiano IRCCS, Piancavallo (VB), Italy; 3 Department of Psychology, Catholic University of Milan, Milan, Italy; University of Sao Paulo, Brazil

## Abstract

A possible link between fibromyalgia (FM) and obesity has been recently suggested but very scanty data on the prevalence of FM in obese populations are available. The aims of the present cross-sectional study were: 1) to estimate the prevalence of FM in a population of obese patients undergoing rehabilitation and 2) to investigate the effect of FM on obese patients' functional capacities. One hundred and thirty Italian obese (Body Mass Index, BMI ≥30) patients admitted to hospital for 1-month rehabilitation treatment took part in the study. All participants were interviewed by a rheumatologist according to the 2010 American College of Rheumatology (ACR) diagnostic criteria for FM. At admission and discharge from hospital (on average, after 28 days), the following measures were compared between the group of patients with FM and the other patients: body weight, body mass index, functional independence (FIM), obesity-related disability (TSD-OC), self-reported functioning and the Timed-Up-Go (TUG) test. Thirty seven patients out of 130 fulfilled the diagnostic criteria for FM. The prevalence rate was 27.7% (95% CI: 20 to 35.4). Between-group comparisons showed that FM patients had higher disability level at the first assessment, had lower scores on the FIM at the final assessment, scored lower on self-reported functioning both at the first and the final assessments and had a lower body weight. The prevalence of FM in our study is much higher than the rates reported in the general normal-weight population (on average, 3.5%) and the 5.15% rate previously reported in a bariatric population. Functional data showed that the FM obese group yielded lower performance capacity and higher disability level as compared to the non-FM obese group. However, due to the relatively small sample size and the selected population, such results need to be confirmed in larger obese subpopulations.

## Introduction

Fibromyalgia (FM) is a chronic disorder of uncertain aetiology, where genetic factors may play a role [1], characterized by widespread pain, muscle tenderness, and decreased pain threshold to pressure and other stimuli.

The 1990 American College of Rheumatology (ACR) criteria for the classification of FM began an era of increased recognition of the syndrome and included widespread pain of at least 3 months' duration and tenderness on pressure at 11 or more of 18 specific tender points [2]. However, most patients with FM also experience other complaints, such as fatigue after mild efforts, morning stiffness, sleep disturbances (non-restorative sleep and insomnia), cognitive disturbances (especially loss of memory), irritable bowel syndrome, headache, paraesthesia, increased lifetime psychiatric comorbidity, most commonly depression and anxiety disorders [3], [4].

Over time, a series of objections to the ACR classification criteria arose: the tender point count was rarely performed in primary care where most FM diagnoses occurred and many physicians did not know how to examine for tender points. Consequently, FM diagnosis in practice has often been a symptom-based diagnosis.

Also, patients who improved or whose symptoms and tender points decreased could fail to satisfy the ACR 1990 classification definition, and the ACR classification criteria set such a high bar for diagnosis that there was little variation in symptoms among fibromyalgia patients.

The diagnostic criteria later suggested by Wolfe et al [5] are not meant to replace the ACR classification criteria. Instead, they were designed to address the above mentioned objections with increasing recognition of the importance of cognitive problems and somatic symptoms.

The development of the 2010 ACR criteria and their modification for survey research [6] made it possible to conduct population-based research relating to FM because the high costs and difficulties surrounding the tender point count ascertainment required by the ACR 1990 criteria were eliminated [7].

### Prevalence of fibromyalgia in the general population

FM occurs in approximately 2% (95% confidence intervals [CI]: 1.4 to 2.7) of the general population in the United States, more common in women than in men, affecting 3.4% of women and 0.5% of men and increased with age [8]. According to a Canadian community survey, FM affects 3.3% (95% CI: 3.2 to 3.4) of adults, with a female-to-male ratio of roughly 3 to 1 [9]. A recent study provided estimates of FM prevalence in the general population of 5 European countries using the London Fibromyalgia Epidemiological Study Screening Questionnaire and a clinical examination by a rheumatologist to confirm or exclude the FM diagnosis according to the 1990 ACR classification criteria. The authors found overall prevalences of FM in the Spanish (2.3%, as reported by [10]), German (3.2%), Portuguese (3.6%) and Italian (3.7%, as reported by [11] and 2.2% by [12]) general populations comparable to those already published, with a slightly lower prevalence in the French population (1.4%). FM was roughly twice as prevalent in females as in males. It appears to be a common syndrome in these 5 Western European countries since a point prevalence of 2.9% would translate to approximately 6 million Europeans affected by FM [11]. The prevalence of FM was found to be as low as 0.8% in Finland [13] and 0.7% in Denmark [14]. Wolfe et al. [15] studied 2,445 subjects randomly selected from the German general population using the ACR 2010 Preliminary Diagnostic Criteria for FM, as modified for survey research, together with the polysymptomatic distress scale and the Patient Health Questionnaire series, and measures of symptoms and quality of life with the European Organization for Research and Treatment of Cancer questionnaire. They found a prevalence of FM of 2.1% (95% Confidence Interval (CI): 1.6 to 2.7), with 2.4% in women (95% CI: 1.5 to 3.2) and 1.8% in men (95% CI: 1.1 to 2.6), but the difference was not statistically significant. Prevalence rose with age.

### Musculoskeletal pain in the obese population

Obesity is a complex disease, defined as a condition of excessive fat accumulation in adipose tissue [16]. Literature evidences show that obese individuals complain more of musculoskeletal pain and physical dysfunction than people of normal weight [17]–[19]. Obesity seems to be associated with some rheumatologic conditions, the most significant association being with knee osteoarthritis [20], [21] but not with osteoarthritis in other weight-bearing joints such as the hip or ankle [22]. There are evidences of a possible link between obesity and osteoarthritis of non-weight-bearing joints, such as hand osteoarthritis [23]. Also carpal tunnel syndrome and low-back pain have a strong association with obesity. In a number of case–control studies, obesity was strongly associated with carpal tunnel syndrome and was independent of diabetes mellitus as a risk factor [24]–[26]. Despite some evidence [27], the literature associating obesity and low-back pain is limited. In a very large population study, obesity was found to be moderately positively associated with recurrent or chronic low-back pain [28].

### Fibromyalgia and obesity

Ursini et al. [29] recently reviewed the scientific evidence about a possible link between FM and obesity, finding an epidemiological correlation: a prevalence of obesity in FM patients of about 40% and a prevalence of overweight of about 30% [30]–[34]. Obesity can be considered an aggravating comorbid condition, affecting negatively FM severity, global quality of life, fatigue, and physical dysfunction. Okifuji et al. [34] found that obesity in FM patients was associated with greater pain sensitivity, poorer sleep quality, and reduced physical strength and flexibility, suggesting that obesity may aggregate FM and weight management may need to be incorporated into treatments for FM. Other recent data have shown that obese patients have an increased risk of developing FM, especially if physically inactive [35]. Saber et al. [36] reported, in a series of 194 patients undergoing bariatric surgery for morbid obesity, a FM prevalence of 5.15%. They also documented the effect of bariatric surgery on FM symptoms: BMI significantly decreased from preoperative to postoperative (from 49.4 to 29.7) and this significant weight loss was associated with statistically significant decrease in median of pain scores (from 9.0 to 3.0), in median tender points (from 18.0 to 3.5) and with a significant quality of life improvement. FM is also linked to obstructive sleep apnea syndrome, a condition typical of obese patients [37]. Clinically, obesity seems to be correlated with some clinical features of FM. Yunus et al. [38] evaluated 211 female patients with FM and found a significant correlation between BMI and Health Assessment Questionnaire scores. Neumann et al. [32] examined the relationship between BMI and measures of tenderness, quality of life, and physical function in 100 female FM patients. In this study, BMI was negatively correlated with quality of life and tenderness threshold and positively correlated with physical dysfunction and point count.

Both FM and obesity are characterized by abnormal cytokine profile. Bazzichi et al. [39] and Wang et al. [40] found elevated levels of IL-8 and TNF-alpha in FM patients. Okifuji et al. [33] found that BMI was positively associated with IL-6 and epinephrine. Weaker relationships were also observed with cortisol and CRP.

In order to further understand the link between obesity and FM, the present cross-sectional study aimed to: 1) estimate the prevalence of FM in a population of obese patients undergoing hospital-based comprehensive rehabilitation and 2) investigate the effect of FM on their functional capacities.

## Methods

### Participants

From February to July 2013, obese patients referred to our Rehabilitation Unit of the San Giuseppe Hospital, Istituto Auxologico Italiano, for a 4-week comprehensive rehabilitation program and weight loss management, were screened for FM according to the 2010 ACR diagnostic criteria (5). Criteria for being admitted to inpatient rehabilitation were: 1) obesity (BMI ≥30), 2) clinical stability and absence of major cardio-respiratory conditions that may contraindicate the rehabilitation process, 3) presence of ostheoarticular conditions affecting the lower limbs (i.e. ostheoartrosis at knee, hip and spine level) with an impact on the capacity to perform activities of daily living and quality of life, as assessed by an obesity-specific disability scale [41]. One hundred and thirty Italian obese inpatients took part in the study. Mean age was 65.3 (SD  = 10.4) and 78.5% were women. At baseline, 15.4% of participants were in obesity class I, 37.7% in class II and 49.9% in class III, according to WHO classification. Mean BMI was 40.2 (see Table 1 for further descriptive details).

**Table 1:** Sample characteristics.

	N	Mean	SD	Median	Min	Max
Age	130	65.3	10.4	66	24	87
BMI	130	40.2	5.8	39.4	30.1	59.5
Body Weight at baseline	130	103.1	17.8	100.9	70.4	173.5

### Procedure

Few days after admission to the rehabilitation unit, participants were interviewed by a rheumatologist according to the 2010 ACR diagnostic criteria for FM [5]. Patients affected by disorders that would have otherwise explained the pain were not diagnosed with FM and were thus included in the comparison group together with all other inpatients that were below the diagnostic thresholds. Those inpatients with significant pain showed rheumatic conditions in inflammatory phase or had been referred for post-surgical rehabilitation after joint replacement. Exclusion of those inpatients was therefore based both on clinical examination, duration and consistency of the symptoms, and the evaluation of the articular damage as observed on X-rays.

### Ethics statements

The study was approved by the Ethics Research Committee of the Istituto Auxologico Italiano. Written informed consent was obtained by all individuals that contributed to the present study.

### Measurements

At admission and discharge from the rehabilitation unit (on average, after 28 days of hospital stay), the following outcome variables were measured: body weight (Kg), BMI (Kg/m^2^), functional independence obesity-related disability and functioning. The Functional Independence Scale (FIM) was used to assess the individuals' level of independence [42] and was administered by a physiotherapist. The scale includes 18 items, each of them is scored from 1 to 7, where 1 represents total dependence and 7 complete independence. Possible scores range from 18 to 126. The 13 physical items can be scored separately from the 5 cognitive items.

The TSD-OC was used to assess obesity-related disability and was administered by a physiotherapist. It is composed of 36 items divided into 7 sections (pain: 5 items; stiffness: 2 items; ADL and indoor mobility: 7 items; housework: 7 items; outdoor activities: 5 items; occupational activities: 4 items; social life: 6 items), which reflect the domains in which individuals experience the most common problems. Individuals are requested to provide a subjective assessment of their disability for each item on a 0–10 mm visual analogue scale, where 10 indicates the highest level of disability and 0 no difficulties in performing the task. A disability score has been defined as (raw total score/max total score)*100.

The Functional Visual Analogue Scale (VAS) (0–10 mm) was used to measure subjective perception of functioning. Patients were also asked to perform a Timed Up & Go (TUG) test [43], [44]. The original purpose of the TUG test was to assess basic mobility skills of frail elderly patients [43] and then adapted by Podsiadlo [44]. This last version consisted of a measurement of the time in seconds for the patient to rise from sitting from a standard arm chair, walk 3 meters, turn, walk back to the chair, and sit down.

### Statistical Analysis

Analyses were performed using the 20th version of the Statistical Package for Social Sciences (SPSS Inc.). Given the strong imbalance between the group of patients with a 2010 ACR diagnosis of Fibromyalgia (n = 36) and the rest of the sample (n = 94), the non-parametric Mann-Whitney test with Monte Carlo approximation was used to make reliable comparisons on the study measures [45]. Cohen's r [46] was used as a measure of FM effect size (r<0.10 “negligible”; 0.10<r<0.30 “small”; 0.30<r<0.50 “Moderate” and r>0.50 “High”). The formula for calculating r is r  =  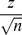 where z is the result of the approximation of the Mann-Whitney U to the normal distribution and n is the sample size [47]. Critical alpha was set at 0.05 and p values ≤0.05 were regarded as statistically significant. Even if multiple tests were performed, no adjustment was made to alpha because the study was not confirmative but explorative. Nonetheless, the inflation of the Type I error rate remains and statistical results should thus be considered with caution.

## Results

Thirty-seven obese patients out of 130 fulfilled the 2010 ACR diagnostic criteria for FM. More than half of participants (59.2%) had a co-morbidity that could have clinically accounted for muscle pain. The prevalence rate of FM was 27.7% (95% CI: 20 to 35.4). Statistical comparisons between patients with a diagnosis of FM and the rest of the sample showed significant differences in some study measures (Table 2). In particular, patients with a diagnosis of FM scored significantly higher on the TSD-OC at the first assessment (p = .001, r = 0.25), had lower scores on the FIM Total at the final assessment (p = .034, r = 0.128) and on the functional VAS both at the first and the final assessments (p = .023, r = 0.138 and p = 0.032, r = 0.12 respectively), and had a lower body weight (p = .001, r = 0,187 at first assessment and p = .002, r = 0,181 at the final assessment). The significant between-group difference in FIM median scores was mainly due to the motor component for which the p-value was almost significant, while the p-value for the cognitive component was largely over the critical alpha and the effect size was negligible (Table 2).

**Table 2:** Between-group comparisons.

Study measures	Non-Fibromyalgia group						Fibromyalgia group							
	N	Mean	Median	SD	Min	Max	N	Mean	Median	SD	Min	Max	p a	r
Age	94	65.6	66	11.0	24	87	36	64.3	64.5	8.7	45	82	.266	0.053
BMI	94	40.7	40.04	5.6	30.1	59.54	36	38.7	37.9	5.9	30.2	55.5	.055	0.067
Body Weight baseline	94	105.9	102.8	18.2	70.4	173.5	36	95.8	93.8	14.6	71.4	133.3	.001	0.187
Body Weight final b	94	102	99.6	17.3	68.3	168	36	92.4	90.4	14.3	69.3	128.5	.002	0.181
Body Weight change	94	−3.9	−3.5	2	−14	0.2	36	−3.4	−3	1.2	−6.2	−1.8	.236	0.006
FIM Motor baseline	94	80.2	83	10.3	32	91	36	77.7	82	10.3	48	88	.096	0.089
FIM Motor final b	91	85	87	8.5	47	91	35	83.6	86	6.9	61	91	.051	0.115
FIM Motor change	90	4.5	4	3.8	0	17	35	6.1	4	5.9	0	27	.236	0.042
FIM Cognition baseline	94	34.7	35	1	29	35	36	34.7	35	0.7	32	35	.770	0.034
FIM Cognition final b	91	34.8	35	0.7	30	35	35	34.8	35	0.6	33	35	.456	0.055
FIM Cognition change	91	0.15	0	0.9	−3	6	35	0.03	0	0.2	0	1	.524	0.044
FIM Total baseline	94	114.9	117.5	11	61	126	36	112.5	116	10.3	82	123	.094	0.093
FIM Total final b	91	119.8	122	8.9	82	126	35	118.5	121	7	96	126	.034	0.128
FIM Total change	91	4.85	4	4.3	0	21	35	6.2	4	5.6	1	25	.250	0.041
TSD-OC Disability Score baseline	90	59.1	63.9	20.5	4.4	90.31	36	70.5	73.8	16	35.8	97	.005	0.229
TSD-OC Disability Score final b	88	36.2	32.5	23.5	0	90.8	35	43.8	48.1	19.4	4	79.7	.087	0.114
TSD-OC Disability Score change	89	−22.8	−23.6	15.8	−67.9	17.5	36	−27.9	−27.9	15.6	−69.4	−1.9	.136	0.069
VAS Function baseline	94	46.9	50	21.9	0	90	36	37.5	42.5	25.7	0	90	.023	0.138
VAS Function final b	92	74.2	75.5	17.9	20	100	35	65.9	68	22.5	5	100	.032	0.120
VAS Function change	92	27.8	25	19.5	−3	90	35	29.4	30	23.4	−20	80	.726	0.031
TUG baseline	85	17.1	15	9.4	5.4	75	33	18.1	15	10.6	8.3	64	.814	0.022
TUG final b	84	13.9	11.85	11.5	4	90	34	14.5	12	8.2	7	40.1	.763	0.031

Notes:

a Based on 10000 sampled tables with starting seed 1585587178.

b Final data were collected at the end of the 28-day in-hospital rehabilitation period.

## Discussion

Obesity represents a real health problem coexisting with FM. Several mechanisms have been proposed to explain “the hidden link” between obesity and FM but whether obesity is a cause or a consequence of FM is difficult to ascertain. Among the mechanisms proposed there are: reduced physical activity, sleep disturbances, depression, dysfunction of thyroid gland and dysfunction of the GH/IGF-1 axis. Obesity may affect sensitivity to noxious stimuli via obesity-related alteration in the endocrine and opioid systems. Okifuji et al. [33] reported that obesity in FM is related to greater levels of proinflammatory indices involved in central sensitization and to the development of chronic latent hyperalgesia in muscles [48]. The most plausible explanation is that all these factors contribute to determine the obese phenotype of many patients and that obesity contributes to perpetuate and worsen the severity of FM [29].

The main aim of our study was to estimate the prevalence of FM in a population of obese patients undergoing hospital-based comprehensive rehabilitation. Results show a prevalence rate (27.7%) much higher than the rates reported in the general normal-weight population (on average, 3.5% from general population studies). The prevalence rate of FM in our sample of obese inpatients is much higher even than the one (5.15%) reported by Saber et al. [29] in a comparably sized bariatric sample. However, sample and methodological differences between the latter and our study are present since the Saber study considered obese patients undergoing bariatric surgery and used a clinical method to diagnose FM, while we adopted both the ACR diagnostic criteria and clinical examination.

To our knowledge, this is the first study that has investigated the prevalence of FM in a sample of obese patients undergoing hospital-based comprehensive rehabilitation by means of the 2010 diagnostic criteria recommended by the ACR. Previous studies have investigated the relationship between FM and obesity in the multiple domains relevant to FM such as pain, hyperalgesic response, function, mood and sleep [34], and the effect of bariatric surgery on FM symptoms in a bariatric sample [36]. Okifuji [34] concluded that obesity in FM adversely affects both the quality and quantity of sleep, physical strength and flexibility, and pain sensitivity to pressure, particularly in the lower body.

As for the effect of FM on body weight and BMI, our data show that the group of inpatients with FM and the group of inpatients without FM differed significantly in body weight, with the former one showing lower values, but not in BMI, for which the effect size was negligible. According to the obesity-FM link hypothesis [29], we might have expected higher weight and BMI values in the FM group. Our findings could have been affected by a probable sampling bias due to convenience recruitment of patients and the relatively small sample size.

Functional data, as measured by the TSD-OC and the VAS, show that the group of FM patients yielded lower performance capacity and higher disability level as compared to the non-FM obese group, with the higher difference observed in the latter variable. It is known that obesity has a profound relationship with disability and, at severe levels, it is *per se* disabling in terms of mobility and exercise capacity [18]. Excessive body weight affects balance capacity [49], muscle strength [50], flexibility [51], walking capacity [52], velocity of locomotion and exhaustion time [53] and, overall, disability and quality of life [41].

In lean subjects, FM is known to have negative repercussions on work ability, performance of daily functional activities [54] and quality of life [55]. Previous studies have demonstrated that functional capacity is compromised in lean FM patients [56], [57], who walk shorter distances in the 6-minute walking test when compared to healthy individuals.

The relationship between BMI, fat mass and lean mass with impairment in different quality of life dimensions in patients suffering from FM has been previously described [58]. The authors found a decrease in quality of life scores, as assessed by the SF-36, for physical functioning, social functioning, emotional role and mental health, in overweight and obese FM patients, with the higher difference observed in emotional role.

In our study, the disability and functional improvements observed at post-rehabilitation were not significantly different between the group of FM patients and the rest of the sample. However, the FM group had significantly lower scores at baseline and the respective functional and disability improvements cannot be directly compared to the ones observed in the group of patients without FM. It is worth specifying that, during the hospital stay, all patients maintained their usual pharmacological therapy, including eventual pain killer medications, while patients diagnosed with FM did not undergo any new specific pharmacological treatment.

Possible limitations of our study are: 1) low generalizability of results to the general obese population; 2) the lack of a comprehensive set of outcome measures including metabolic and psychological indexes;3) the semi-quantitative nature of the TSD-OC scale as well as the subjective nature of the interview that may have generated false positive results in a population with potential psychological or psychiatric confounding factors.

Our results are not conclusive. It should be borne in mind that the concept of a “general obese population” implies a wide range of BMIs, comorbidities, degree of disabilities and, ultimately, an extremely heterogeneous population. Further studies should therefore estimate the prevalence of FM in different obese subpopulations (i.e., “healthy” obese or obese with other comorbidities) and assess its effect on participants' functional capacities as well as disability levels. It seems important for rehabilitation of obese patients to investigate in depth those effects since the presence of FM may lower functional capacities, increase self-perceived disability and, ultimately, require additional rehabilitative resources.
